# Prevalence of c-*myc* expression in breast lesions associated with microcalcifications detected by routine mammography

**DOI:** 10.1590/S1516-31802009000200003

**Published:** 2009-07-06

**Authors:** Renato Coimbra Mazzini, Simone Elias, Afonso Celso Pinto Nazário, Cláudio Kemp, Ângela Flávia Logullo

**Affiliations:** 1 MD. Physician in the Breast Group of the Department of Gynecology, Universidade Federal de São Paulo - Escola Paulista de Medicina (Unifesp-EPM), São Paulo, Brazil.; 2 MD. Physician in the Department of Gynecology, Universidade Federal de São Paulo - Escola Paulista de Medicina (Unifesp-EPM), São Paulo, Brazil.; 3 MD, PhD. Chief professor of Gynecology, Mastology Sector, Department of Gynecology, Universidade Federal de São Paulo - Escola Paulista de Medicina (Unifesp-EPM), São Paulo, Brazil.; 4 MD, PhD. Associate professor, Department of Gynecology, Universidade Federal de São Paulo - Escola Paulista de Medicina (Unifesp-EPM), São Paulo, Brazil.; 5 MD, PhD. Associate professor, Department of Pathology, Universidade Federal de São Paulo - Escola Paulista de Medicina (Unifesp-EPM), São Paulo, Brazil.

**Keywords:** Proto-oncogene proteins c-myc, Gene expression, Mammography, Breast neoplasms, Biopsy, needle, Proteínas proto-oncogênicas c-myc, Expressão gênica, Mamografia, Neoplasias mamárias, Biópsia por agulha.

## Abstract

**CONTEXT AND OBJECTIVE:**

Genetic abnormalities in cell proliferation-regulating genes have been described in premalignant lesions. The aims here were to evaluate c-myc protein expression in non-palpable breast lesions associated with microcalcifications, detected by screening mammography, and to compare these results with histopathological, clinical and epidemiological variables.

**DESIGN AND SETTING:**

Analytical cross-sectional study, with retrospective data collection, in a university hospital in São Paulo.

**METHODS:**

Seventy-nine female patients who underwent routine mammography between 1998 and 2004 were studied. Lesions classified by the Breast Imaging Reporting and Data System (BI-RADS) as 4 or 5 underwent percutaneous biopsy using a large-core needle. Ninety-eight lesions were studied anatomopathologically. Paraffin blocks properly representing the lesions were selected for immunohistochemical analyses using the streptavidin-biotin-peroxidase technique with monoclonal mouse c-myc antibodies.

**RESULTS:**

Among the 98 lesions, 29 (29.6%) contained malignant neoplasia; 40 (40.8%) had a positive immunohistochemical reaction for c-myc. When the groups were divided between lesions without atypias versus atypical lesions plus malignant lesions, 31.03% of the 58 lesions without atypias were positive for c-myc and 55% of the 40 malignant and atypical lesions (P = 0.018). Comparing the atypical lesions with ductal carcinoma *in situ* versus the benign lesions without atypias, c-myc was present in 51.61% of the 31 atypical lesions and 31.03% of the benign lesions without atypias (P = 0.057).

**CONCLUSION:**

C-myc protein was more frequently expressed in atypical and malignant lesions than in benign lesions without atypias. C-myc expression correlated with the presence of atypias (P = 0.018).

## RESUMO

**CONTEXTO E OBJETIVO:**

Alterações nos genes reguladores da proliferação celular foram descritas em lesões pré-malignas. Os objetivos foram avaliar a expressão da proteína c-myc em biópsias de lesões mamárias não-palpáveis associadas a microcalcificações detectadas em mamografias de rastreamento e comparar estes resultados com as variáveis histopatológicas, clínicas e epidemiológicas.

**DESENHO E LOCAL:**

Estudo retrospectivo, em um hospital universitário em São Paulo.

**MÉTODOS:**

Setenta e nove pacientes do sexo feminino submetidas a mamografia de rotina de 1998 a 2004 foram estudadas. As lesões classificadas pelo sistema BI-RADS (Breast Imaging Reporting and Data) como 4 e 5 sofreram biópsias percutâneas com agulha grossa. Do ponto de vista anatomopatológico, foram estudadas 98 lesões. Os blocos com representação adequada para estudo imunoistoquímico com a técnica da estreptoavidina-biotina-peroxidase com o anticorpo monoclonal de camundongo c-myc foram incluídos.

**RESULTADOS:**

Das 98 lesões, 29 (29,6%), continham neoplasia maligna; 40 (40,8%) tiveram reação de imunoistoquímica positiva para o c-myc. Quando divididos os grupos em lesões sem atipia versus lesões atípicas mais lesões malignas, encontramos o c-myc positivo em 31,03% das 58 lesões sem atipias e 55% das 40 lesões atípicas e malignas (P = 0,018). Quando agrupamos as lesões atípicas com o carcinoma ductal *in situ* (CDIS) *versus* as lesões benignas sem atipias, observamos a presença do c-myc em 51,61% das 31 lesões atípicas e 31,03% das lesões benignas sem atipias (P = 0,057).

**CONCLUSÃO:**

A proteína c-myc está mais frequentemente expressa em lesões atípicas e malignas do que em lesões benignas sem atipia. A expressão do c-myc está correlacionada com a presença de atipia (P = 0,018).

## INTRODUCTION

At present, the best screening method for breast cancer is routine annual mammography examinations for women over 40 years of age. This has been correlated with a reduction of up to 30% in the mortality rates due to breast cancer, around the world. Screening using mammography is an extremely sensitive method for detecting both malignant and benign breast lesions, and the great majority of breast lesions seen on mammographs are non-palpable. The high sensitivity of the method is largely due to findings of microcalcifications associated with the lesions. Areas of microcalcifications often need to be investigated by means of biopsies. Today, 10 to 40% of biopsies on areas of microcalcifications find malignant lesions.^1^^,^^2^^,^^3^^,^^4^ Precursor lesions or even minimally invasive lesions associated with these microcalcifications provide greater accuracy in identifying lesions at early stages. Microcalcifications have come to represent 15 to 20% of the breast radiographic abnormalities diagnosed in screening tests.^5^^,^^6^^,^^7^

Today, there are several methodological approaches for performing breast biopsies. The gold standard is still surgical removal of the lesions by means of open surgery.^4^ However, less invasive methods consisting of percutaneous biopsy with a large-core needle, by means of both automatic propulsion (core biopsy) and vacuum assistance (“mammotomy”), are increasingly used.^8^ The disadvantage of these less invasive types of approach is that they sometimes do not remove the lesions, which leads to the need for follow-up regarding the patient’s remaining microcalcifications.^9^

Genetic abnormalities in cell proliferation-regulating genes have been described in premalignant lesions.^10^ Among the oncogenes connected with controlling the cell cycle, the role of the oncogene *c-myc* stands out. Since its discovery by Bishop, in the 1970s, its central role in the proliferation and malignant transformation of animal tissue has been widely demonstrated.^11^

One important carcinogenesis route that has been studied in relation to breast cancer is the Wnt pathway. The first Wnt gene was cloned from the rat genome in 1982. This family produces an enormous quantity of proteins that are related to various normal cell functions, from differentiation and proliferation to cell survival capacity.^12^ The best known and most studied are Wnt itself (described in Wilms tumors), adenomatous polyposis coli (APC) (responsible for hereditary colon polyposis syndrome), catenin (a protein for cell support and connection with the basal membrane) and T-cell transcription factor (TCF). There is growing evidence that the proteins in the Wnt signaling pathway and/or its components have an extensive relationship with human breast cancer.^13^ Because this is a complex pathway for activating and producing cell proteins, it acts in reactions that include several oncogenes. One of the target genes that have been described is *c-myc*.^14^ For this reason, c-myc protein expression is evaluated as an indirect marker for this pathway in breast carcinogenesis.

The c-myc protein acts as a transcription factor, i.e. a protein that is capable of binding to specific deoxyribonucleic acid (DNA) sites with the purpose of modulating the gene transcription. Expression of the *c-myc* gene is unregulated in several types of human neoplasia. Contrary to other oncogenes such as those of the ras family, activation of *c-myc* expression does not require mutations or changes in amino acid sequences.

The mechanisms through which the functions of the *c-myc* gene can be altered include action by viral particles; amplification of the locus on the chromosome, such as in neuroblastomas (N-*myc*) and pulmonary neoplasia (L-*myc*); chromosome translocations, such as in Burkitt’s lymphomas; or overexpression of the protein due to gene or transcription factor mutations. A gene regulation pathway for the *c-myc* gene through the expression line of the Wnt gene has been described. By means of the intermembrane receptor, the Wnt gene acts on the protein APC and the latter binds to catenin to form a complex in the cytoplasm. When this complex is not formed, which could be through changes in the action of the Wnt gene, APC or catenin, the latter actively moves to the nucleus and acts as a transcription factor for the *c-myc* gene.^14^

Under physiological conditions, the main role of *c-myc* is to promote cell replication, in response to extracellular signals that arouse the cell cycle from rest.^15^ It is now known that *c-myc* participates in the majority of cell functions, for example replication, growth, metabolism, differentiation and apoptosis.^16^^,^^17^^,^^18^^,^^19^^,^^20^ Carcinogenesis relating to *c-myc* occurs when this is expressed aberrantly through amplification or genetic alteration, thus leading to events that go from promotion of excessive proliferation of altered cells to inhibition of apoptosis.^21^^,^^22^ Amplification and/ or overexpression of this gene have been described in almost all types of malignant neoplasia in human beings.^23^

Amplification of the c-myc oncogene has already been described in benign breast lesions such as fibroadenoma.^11^ We do not know of any reports on abnormalities in premalignant lesions associated with microcalcifications that were found by means of screening mammography.

## OBJECTIVE

Our objective here was to evaluate the prevalence of c-myc protein expression in non-palpable breast lesions associated with microcalcifications that underwent biopsy following detection by means of screening mammography, and to compare these results with histopathological variables.

## MATERIALS AND METHODS

### Patients and design

This study was granted prior approval by the Research Ethics Committee of our university hospital. Ninety-eight lesions among 79 female patients who were admitted to our service between 1998 and 2004 were studied. All these patients had undergone routine mammography, were found to present microcalcifications and were classified as 4 or 5 using the 2003 version of Breast Imaging Reporting and Data System (BI-RADS 2003).^24^ The patients who were included underwent percutaneous biopsies with a large-core needle, using the Mammoguide^®^ system (Philips MammoDiagnost MD 4000 mammograph, Philips Medical Systems BV, Netherlands) as the guide for image and biopsy site identification. The techniques used were stereotaxis-guided mammotomy and core biopsy with a large-core needle of caliber 12 G, adapted to an automatic propulsion system with a 2.2 cm cursor (Biopty, Bard Urological, Covington, Georgia, United States).

### Histological analysis

The samples were fixed in 10% formalin, labeled with the patient’s name and sent to the pathology laboratory of the Universidade Federal de São Paulo - Escola Paulista de Medicina (Unifesp-EPM). After processing and embedding in paraffin, five-micron sections were mounted on slides and stained with hematoxylin and eosin using the recommended technique.

All the cases included in our study were reviewed from an anatomopathological point of view, by two independent pathologists. The diagnoses were classified by consensus as benign entities without atypias, premalignant or atypical entities (atypical hyperplasia) or malignant entities.

### Immunohistochemical analysis

For the immunohistochemical analysis, new five-micron copies from the blocks studied were mounted on blank silanized slides. The faithfulness of all the reactions was tested by comparing each of them with positive and negative controls that had previously been established, which was done concomitantly. All the steps were preceded by two five-minute baths in phosphate-buffered solution (PBS) at pH 7.4 (except for the secondary antibody).

The *c-myc* expression was analyzed according to its distribution and positivity pattern. It was analyzed in the epithelial component of the tumors. When c-myc protein was found to be present in predominantly nuclear locations in more than 10% of the neoplastic cells, this was taken to be a positive finding.

The results obtained were subjected to statistical analysis using descriptive and analytical techniques. The chi-squared univariance test was performed (in 2 x 2 tables), and when the frequency was less than five, the Fisher test was used. P-values of less than or equal to 0.05 were taken to be significant.

## RESULTS

Among the 98 lesions studied, nine had a diagnosis of invasive ductal carcinoma (IDC), 20 were ductal carcinoma *in situ* (DCIS), 11 were atypical ductal hyperplasia (ADH) and 58 were benign lesions without atypias, such as fibrocystic abnormalities, fibrosclerosis, adenosis and lesions with focal hyperplasia without atypias.

The *c-myc* antigen was more frequently expressed in cases of IDC and ADH (Table 1). Comparing the atypia cases (ADH) with the benign cases, it was seen that negative findings for c-myc were predominant in the benign cases, and that this was statistically significant (P = 0.039; Table 2). The benign cases also seemed to be associated with negative c-myc findings when compared with the IDC cases and IDC plus DCIS (malignant) cases, although without statistical significance (P < 0.061).

Finally, dividing the lesions into two groups, the first containing the lesions with atypias and the neoplastic lesions (IDC and DCIS) and the second containing the lesions without atypias, the *c-myc* expression rates were 55% and 31.03%, respectively. We observed a greater frequency of *c-myc* in the lesions with atypias, and this difference was statistically significant (P = 0.018), as shown in Table 3.

**Table 1.** Expression of the c-*myc* antigen according to the histopathological classification of breast lesions

Histopathological results	n	**Positive for c-*myc***	%
Invasive ductal carcinoma	9	6	66.7
Ductal carcinoma *in situ*	20	9	45
Atypical ductal hyperplasia	11	7	66.7
Benign lesions without atypias	58	18	31
Total	98	40	100

**Table 2.** Comparison between positive and negative c-myc expression and the types of breast lesion, grouped according to benign and malignant histological results

Types of lesion	**c-*myc***		P
	Negative n (%)	Positive n (%)	
Malignant (IDC and DCIS) Benign without atypias	14 (48.3) 40 (69.0)	15 (51.7) 18 (31.0)	0.061
Atypias (ADH) Benign	4 (36.4) 40 (69.0)	7 (63.6) 18 (31.0)	0.039
DCIS Benign without atypias	11 (55.0) 40 (69.0)	9 (45.0) 18 (31.0)	0.258
IDC Benign without atypias	3 (33.3) 40 (69.0)	6 (66.7) 18 (31.0)	0.060^*^

IDC = invasive ductal carcinoma; DCIS = ductal carcinoma in situ; ADH = atypical ductal hyperplasia. p-values obtained using the chi-squared frequency test. *P-value obtained using the Fisher exact test.

**Table 3.** Expression of the c-myc antigen according to the presence or absence of cell atypias

Presence or absence of atypias	Negative for c-myc	%	Positive for c-myc	%	Total
Without atypias With atypias	40 18	68.97 45	18 22	31.03 55	58 40
Total	58		40		98

Pearson chi-squared (1) = 5.6286; P = 0.018.

## DISCUSSION

The hypothetical model for tumor progression in breast carcinogenesis, through a sequential (multistep) chain of events, was put forward by Lakhani in 1999.^25^ Despite the complexity of studying this model for the breast, due to the morphological heterogeneity of preinvasive lesions, this author based his theory on three main sources: experiments on animals, reviews on human histological material and genetic analyses on precursory breast lesions.

In experiments on animals following exposure to the tumor virus MuMTV (murine mammary tumor virus), the normal epithelium was found to be transformed into hyperplastic alveolar nodules that, when transplanted into normal breast tissue, present a higher chance of malignant transformation. In reviews on human histological material, ADH and DCIS were found in specimens of invasive carcinoma, which also showed that these lesions occasionally present morphological transition and continuity with each other. Furthermore, atypical hyperplasia was five times more common in breasts containing malignity than in normal breasts.^25^

Other evidence has come from a review of prospective studies by Page et al.^26^ that found 2.5 times more carcinomas in patients whose previous biopsies showed the usual ductal hyperplasia and five times more when atypical. They found 5.3 times more carcinomas with ADH and 11 times more in conjunction with family histories of cancer. These authors also showed that in breasts with DCIS in biopsies, 30% more invasive carcinomas were found after 6.1 years of follow-up.

To conclude his theory, Lakhani^25^ also reported the various progressive genetic similarities between DCIS and IDC, between ADH and DCIS and between the usual ductal hyperplasia and atypical ductal hyperplasia. In our study, we were also able to observe complexity of proliferative lesions in the same patients. Thus, we found three cases of ADH with DCIS and two cases of DCIS with IDC.

It is known that the carcinogenic process of tumor progression covers a series of intermediate stages, including activation of oncogenes and inactivation of tumor-suppressing genes.^27^^,^^28^ The stages preceding tumor invasion that are described as DCIS and ADH frequently involve several early genetic changes.^29^ These lesions may be found in association with infiltrative neoplasia, which suggests that the cumulative procedure of genetic damage does not always follow a single linear sequence of events.^30^

The activated *c-myc* gene may perform an important biological role in the tumorigenesis process.^31^ One of the most important actions involves the proliferation process. Early induction of *c-myc* may occur through different pathways, involving the transition from the quiescent cellular state (G0) to proliferation (G1). Moreover, *c-myc* is continually expressed in cells undergoing proliferation.^31^ The mechanism for action leading to the entry of cells into the cell cycle involves inactivation of cyclin-kinase antagonist proteins and inactivation of the cyclin E antagonist. The presence of *c-myc* from G1 onwards seems to be essential for the cell cycle sequence.^32^ On the other hand, in cells that enter the differentiation process, the expression decreases and this is important for terminal differentiation. Since *c-myc* expression is also associated with the apoptosis process, lack of control over *c-myc* expression may lead to increased cell death.^31^

In a meta-analysis, Deming et al.^33^ observed that, on average, 15.5% of breast cancer biopsies presented *c-myc* gene amplifications of more than three times normal values. Studies using the immunohistochemical technique have demonstrated that, in 50 to 100% of the cases, there were increased levels of the c-myc protein.^34^ Mai showed that there was a greater percentage of cancer cases with aberrant c-myc protein levels than of cases showing gene amplification. This would also imply that changes in the stability of messenger ribonucleic acid (RNA) or its protein could be the mechanism for increased c-myc levels, which would be consistent with reports that overexpression precedes gene amplification, thereby leading to chromosome instability.^35^

Following this line of reasoning, it might be supposed that findings of c-myc protein in benign lesions, as in our study, could precisely represent this initial step of gene overexpression and/or amplification and the initial step in breast carcinogenesis. In fact, when we compared the presence of *c-myc* in atypical lesions with its presence in benign lesions without atypias, we found a statistically significant difference (P = 0.039). Likewise, dividing the lesions into two groups, such that the first contained the lesions with atypias and the neoplastic lesions (IDC and DCIS) and the second contained the lesions without atypias, the *c-myc* expression was 55% and 31.03%, respectively. Thus, we observed greater frequency of *c-myc* in the lesions containing atypias, and this difference was also statistically significant (P = 0.018).

Even without reaching significance, we could see a certain tendency (P = 0.06) towards *c-myc* expression when the IDC and DCIS results and the results from the benign lesions were analyzed together. This was also seen when we compared the IDC findings alone with the benign lesions (P = 0.06). These results allow it to be presupposed that the presence of *c-myc* may signify increased risk of progression of the benign proliferative lesions, or even of the DCIS.

The number of cases studied did not allow separate identification of the individual pattern of *c-myc* expression in the benign breast lesions, separated according to the relative risk of progressing to invasive carcinoma. Nevertheless, among the adenosis, papilloma and typical ductal hyperplasia cases (lesions that are said to be proliferative and considered to present a low risk of breast cancer), we found six cases that were positive for *c-myc*, out of the 14 studied. On the other hand, among the non-proliferative lesions, such as fibrocystic abnormalities, only one case out of 14 was positive.

We also observed that the pattern of *c-myc* presence was variable and that it could coexist in different lesions in the same patient. For example, in some patients in whom malignant lesions and low-risk benign proliferative lesions (such as adenosis and the usual ductal hyperplasia) coexisted, we found that both types of lesion were positive for *c-myc* (two cases out of six evaluated). This finding reinforces the supposition that the initial lesion (adenosis) had already been amplified by the *c-myc* gene much before the evolution into an atypical proliferative lesion or even into carcinoma. It corroborates the hypothetical model for tumor progression through a multistep sequential chain of events for breast carcinogenesis.^25^

Schmitt and Reis-Filho^36^ reported positive findings of *c-myc* in breast cancer cases, ranging from 1% to 94.4%. This large difference in positive findings of *c-myc* has a variety of reasons. Among these, perhaps the main reason is differences in investigation methods, which may make use of immunohistochemical or hybridization techniques, such as fluorescence in situ hybridization (FISH) and chromogenic *in situ* hybridization (CISH). These latter methods are clearly more sensitive, but much more expensive to perform.

Another reason would be the fact that *c-myc* presence is related to different types of tumor. For example, high nuclear grade tumors have a higher chance of presenting overexpressed proteins than do low grade tumors.^11^

Reinforcing this idea of overexpression of c-myc protein in cells that are more differentiated, Le Roy et al.^37^ demonstrated that patients with breast tumor receptors positive for estrogens who received treatment with tamoxifen showed decreased levels of c-myc messenger RNA, in comparison with patients who had not received this drug. This result suggests that tamoxifen antagonized the effect of estrogens in relation to *c-myc* expression.

It is known that percutaneous biopsies from benign lesions such as typical ductal hyperplasia, florid adenosis, papillomatosis and even atypical ductal hyperplasia result in some cases of underestimated diagnosis. It is also known that, during follow-up, some of these lesions end up evolving into carcinoma. We might therefore suggest that tamoxifen should be used in cases of biopsy findings of benign proliferative lesions that nonetheless are positive for *c-myc*.

The study by Berns et al.^38^ also suggested that there is an inverse relationship between *c-myc* presence and human epidermal growth factor receptor 2 (HER2/neu) presence. This opens up even greater possibilities for future studies on *c-myc* involving possible drug preparations that would be capable of acting directly on amplified c-myc, in the same way as with c-*erb-b2*, such as the use of herceptin and HER2/neu.

Studying the expression of the *c-myc* gene has produced some guidelines regarding carcinomas that may in the future contribute towards more accurately determining tumor behavior, because of the associations of *c-myc* with cell proliferation and chemoresistance.^39^

## CONCLUSION

The c-myc protein is more frequently expressed in atypical and malignant lesions than in benign lesions without atypias. The expression of *c-myc* correlates with the presence of atypias.
